# Creg1 Regulates Erythroid Development via TGF‐β/Smad2‐Klf1 Axis in Zebrafish

**DOI:** 10.1002/advs.202402804

**Published:** 2024-07-02

**Authors:** Xiao Han, Wenxin He, Dongguo Liang, Xiaohui Liu, Jun Zhou, Hugues de Thé, Jun Zhu, Hao Yuan

**Affiliations:** ^1^ Shanghai Institute of Hematology State Key Laboratory of Medical Genomics National Research Center for Translational Medicine at Shanghai Ruijin Hospital Shanghai Jiao Tong University School of Medicine Shanghai 200025 China; ^2^ CNRS‐LIA Hematology and Cancer Sino‐French Research Center for Life Sciences and Genomics Ruijin Hospital Shanghai Jiao Tong University School of Medicine Shanghai 200025 China; ^3^ Université de Paris 7/INSERM/CNRS UMR 944/7212 Equipe Labellisée Ligue Nationale Contre le Cancer Hôpital St. Louis Paris 75010 France

**Keywords:** Creg1, erythropoiesis, Klf1, TGF‐β/Smad2 signaling

## Abstract

Understanding the regulation of normal erythroid development will help to develop new potential therapeutic strategies for disorders of the erythroid lineage. Cellular repressor of E1A‐stimulated genes 1 (CREG1) is a glycoprotein that has been implicated in the regulation of tissue homeostasis. However, its role in erythropoiesis remains largely undefined. In this study, it is found that *CREG1* expression increases progressively during erythroid differentiation. In zebrafish, *creg1* mRNA is preferentially expressed within the intermediate cell mass (ICM)/peripheral blood island (PBI) region where primitive erythropoiesis occurs. Loss of *creg1* leads to anemia caused by defective erythroid differentiation and excessive apoptosis of erythroid progenitors. Mechanistically, *creg1* deficiency results in reduced activation of TGF‐β/Smad2 signaling pathway. Treatment with an agonist of the Smad2 pathway (IDE2) could significantly restore the defective erythroid development in *creg1* ^−/−^ mutants. Further, *Klf1*, identified as a key target gene downstream of the TGF‐β/Smad2 signaling pathway, is involved in *creg1* deficiency‐induced aberrant erythropoiesis. Thus, this study reveals a previously unrecognized role for Creg1 as a critical regulator of erythropoiesis, mediated at least in part by the TGF‐β/Smad2‐Klf1 axis. This finding may contribute to the understanding of normal erythropoiesis and the pathogenesis of erythroid disorders.

## Graphical abstract

This study reveals a previously unknown role of cellular repressor of E1A‐stimulated genes 1 (Creg1) in erythroid development. Creg1 loss causes anemia due to impaired differentiation and excessive apoptosis of erythroid cells. The TGF‐β/Smad2‐Klf1 pathway is crucial for Creg1‐mediated erythropoiesis, shedding light on erythrocyte formation and related disorders.

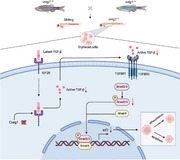

## 1 Introduction

Red blood cells (RBCs), also referred to as erythrocytes, are the most abundant cells in the blood, which are responsible for transporting oxygen and carbon dioxide. In healthy adults, approximately 2 million RBCs are generated each second to maintain the steady‐state normal red cell mass, through the process of erythropoiesis.^[^ ^1^ ^]^ Erythropoiesis is a tightly regulated and multi‐step process originating from a multipotent hematopoietic stem cell (HSC) to a mature, enucleated erythrocyte. Traditionally, this process has been divided into three stages: early erythropoiesis, terminal erythroid differentiation, and reticulocyte maturation.^[^ ^2^ ^]^ During early erythropoiesis, HSCs develop progressively into common myeloid progenitors, megakaryocyte‐erythrocyte progenitors, and then erythroid progenitors, like burst‐forming unit‐erythroid (BFU‐E) and colony‐forming unit erythroid (CFU‐E) cells.^[^ ^3^, ^4^ ^]^ CFU‐E cells then give rise to proerythroblasts, which marks the “beginning” of terminal erythroid differentiation. The proerythroblasts undergo sequential mitosis to produce basophilic, polychromatic, and orthochromatic erythroblasts that enucleate to become reticulocytes.^[^ ^5^, ^6^ ^]^ At the final step of erythropoiesis, reticulocytes mature into discoid RBCs accompanied by the loss of intracellular organelles, a reduction of the cell volume, and extensive membrane remodeling.^[^ ^7^, ^8^, ^9^, ^10^, ^11^, ^12^ ^]^

The zebrafish has emerged as an ideal model organism for the study of erythropoiesis.^[^ ^13^ ^]^ Zebrafish hematopoiesis, similar to mammals, consists of two successive waves, primitive wave and definitive wave. The primitive wave occurs transitorily and produces erythrocytes, macrophages and granulocytes during embryonic development, while the definitive wave generates definitive HSCs to maintain the lifelong production of all lineages of blood cells.^[^ ^14^ ^]^ Unlike similar cells in mammals, zebrafish erythrocytes are nucleated. Despite the differences in erythrocyte morphology, many critical genes and signaling pathways controlling erythropoiesis are well conserved between zebrafish and mammals.^[^ ^13^, ^15^ ^]^

CREG1 is a secreted glycoprotein consisting of 220 amino acids (human isoform), which contains a signal peptide (amino acids 1‐31) and three potential N‐glycosylation sites (N160, N193, and N216).^[^ ^16^, ^17^ ^]^ It was initially described as a transcription repressor that antagonized the transcriptional activation and cellular transformation induced by the adenovirus E1A oncoprotein.^[^ ^18^ ^]^ Later several studies have demonstrated that overexpression of *CREG1* inhibited cellular proliferation and promoted differentiation.^[^ ^16^, ^19^, ^20^, ^21^ ^]^ Conversely, decreased expression of *CREG1* had the opposite effects.^[^ ^20^, ^22^, ^23^ ^]^ CREG1 has also been suggested as a senescence‐related protein which could enhance p16^INK4a^‐induced cellular senescence by transcriptional repression of cell cycle‐regulated genes.^[^ ^24^ ^]^ Additionally, CREG1 exhibited the ability to stimulate brown adipogenesis.^[^ ^25^, ^26^ ^]^ More recently, CREG1 has been shown to be involved in macroautophagy/autophagy modulation.^[^ ^27^, ^28^ ^]^ However, the physiological role of CREG1 in erythropoiesis has not yet been explored. In the present study, we generated a *creg1* knockout zebrafish model to investigate the function of *creg1* during erythroid development and examine the underlying molecular mechanism(s), both in vivo and in vitro.

## 2 Results

### 2.1 The Expression Pattern of *creg1* during Erythropoiesis

**Figure 1:**
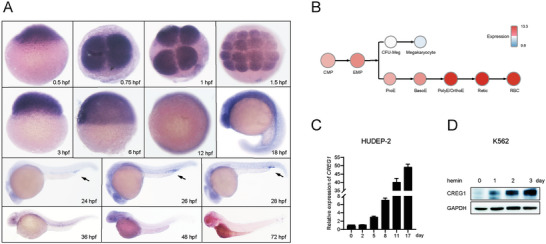
The expression profile of *creg1*. A) WISH assay of *creg1* during early zebrafish embryonic development. The arrows indicate the ICM/PBI region. B) *CREG1* mRNA expression in normal human hematopoietic cells was analyzed using BloodSpot database. CMP, Common myeloid progenitor; EMP, Megakaryocyte/erythroid progenitor; ProE, proerythroblasts; BasoE, basophilic erythroblasts; PolyE/OrthoE, polychromatophilic/orthochromatophilic erythroblasts; Retic, reticulocyte; RBC, red blood cell; CFU‐Meg, Colony Forming Unit‐Megakaryocytic. C) Quantitative PCR analysis of *CREG1* expression during erythroid differentiation of HUDEP‐2 cells. Data shown are the means ± SEM from three independent experiments. D) Western blot analysis of CREG1 in hemin‐treated K562 cells.

Previous studies have shown that *creg1* is broadly expressed in adult mouse tissues, including bone marrow and spleen.^[^ ^16^, ^27^ ^]^ To determine the expression profile of *creg1* during embryogenesis in zebrafish, we conducted whole mount in situ hybridization (WISH) on wild‐type (WT) embryos at multiple developmental stages (**Figure** **1**A). Zebrafish *creg1* was maternally expressed, and the transcript was ubiquitously distributed from the one‐cell stage to the segmentation stage. At 24–28 h post fertilization (hpf), its expression was dominant in the ICM/PBI region where primitive erythropoiesis occurs. From 48 hpf onwards, the expression level was gradually decreased, and became extremely low or undetectable in most tissues by 72 hpf, perhaps because of the limited accessibility of *creg1* RNA probes. Moreover, by interrogating the BloodSpot,^[^ ^29^ ^]^ a database of mRNA expression of hematopoietic cells, *CREG1* expression increased progressively during erythroid differentiation (Figure 1B). The increased expression level of *CREG1* during erythroid differentiation was further validated in HUDEP‐2 cells (Figure 1C), an immortalized human erythroid progenitor cell line,^[^ ^30^ ^]^ and K562 cells (Figure 1D). Therefore, based on the spatial and temporal expression profiles, we speculate that Creg1 may play a regulatory role in erythropoiesis.

### 2.2 Creg1 Regulates Erythroid Development

In order to explore the potential role of *creg1* during the processes of erythroid development, we first utilized antisense oligonucleotide (morpholino, MO)‐mediated gene knockdown strategy in zebrafish. The result showed that the expression level of embryonic globin gene *hbae1* was significantly decreased in *creg1* morphants compared with WT embryos (Figure S1A,B, Supporting Information), indicating that knockdown of *creg1* impairs erythropoiesis. To further confirm this notion, *ο*‐dianisidine staining was performed to investigate the total amount of hemoglobin. Consistently, the stain for hemoglobin was strikingly reduced in the *creg1* MO‐injected embryos (Figure S1C,D, Supporting Information). Thus, these data indicate that loss‐of‐function of *creg1* leads to defective erythroid development in zebrafish.

**Figure 2:**
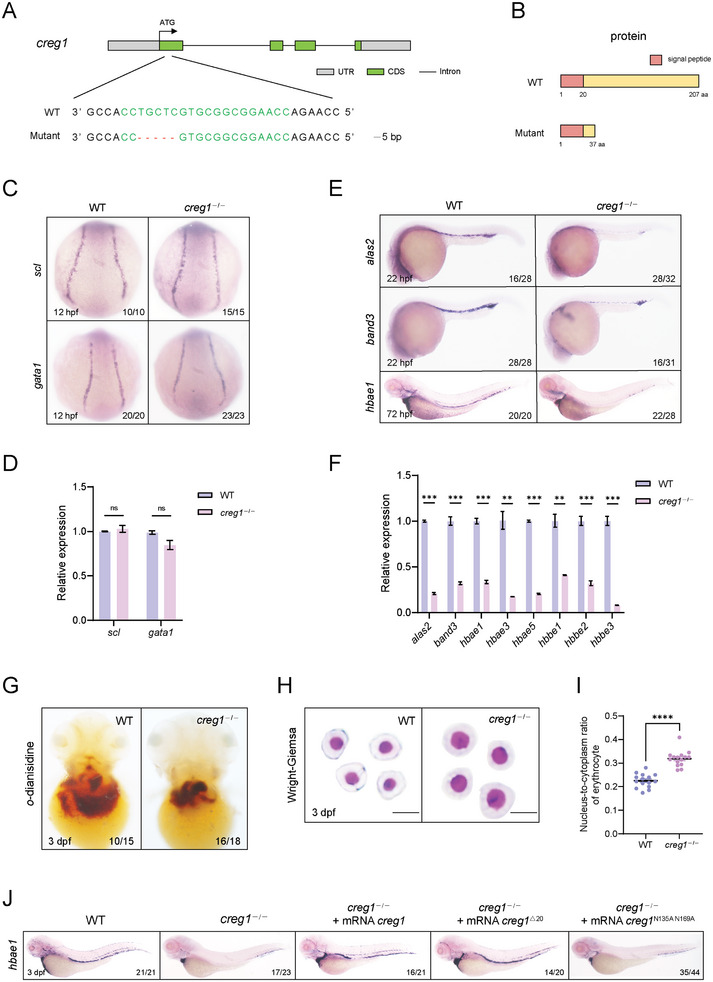
*creg1* deficiency leads to defective erythroid differentiation and maturation. A) Genetic inactivation of the zebrafish *creg1* gene based on CRISPR/Cas9. Schematic representation of CRISPR/Cas9 target site at exon 1 as used in this study. The gRNA target site is highlighted in green, and the indel is indicated by a red dash. B) Schematic representation of the WT and mutant Creg1 proteins. C) WISH assay of *scl* or *gata1* at 12 hpf in WT siblings and *creg1* ^−/−^ mutants. D) Quantitative PCR analysis of *scl* or *gata1* expression at 12 hpf in WT siblings and *creg1* ^−/−^ mutants (*n*=30 embryos per group). E) WISH assay of *alas2, band3* or *hbae1* at 22 or 72 hpf in WT siblings and *creg1* ^−/−^ mutants, respectively. F) Quantitative PCR analysis of *alas2, band3* or *globins* expression at 72 hpf in WT siblings and *creg1* ^−/−^ mutants (*n*=30 embryos per group). G) *o*‐dianisidine staining in WT siblings and *creg1* ^−/−^ mutants at 3 dpf. H) Wright‐Giemsa staining of erythrocytes from WT siblings and *creg1^−/−^* mutants at 3 dpf. I) Nucleo‐cytoplasmic ratio analysis of erythrocytes from WT siblings and *creg1^−/−^* mutants at 3 dpf (*n* = 15 cells per group). J) WISH assay of *hbae1* at 3 dpf for embryos injected with mRNA encoding distinct *creg1* mutants. Data shown are the means ± SEM. Statistical significance was calculated using the Student's *t*‐test. **, *p* < 0.01; ***, *p* < 0.001; ****, *p* < 0.0001; ns, not significant.

To further confirm the obtained results, we generated a stable *creg1* mutant zebrafish line using the CRISPR/Cas9 system. Sanger sequencing revealed a 5 bp deletion in exon 1 of *creg1* gene locus, which resulted in the synthesis of a truncated Creg1 protein lacking the C‐terminal 170 amino acids (**Figure** **2**A,B). In zebrafish, primitive hematopoiesis begins around 12 hpf and produces erythroid and myeloid cells from mesoderm‐derived hemangioblasts.^[^ ^14^, ^31^ ^]^ The erythroid progenitors first arise from the posterior lateral mesoderm (PLM) that forms the ICM later.^[^ ^32^ ^]^ Using in situ hybridization, we observed no significant difference in the expression levels of *scl* (a hematopoietic progenitor marker) and *gata1* (an erythroid progenitor marker) between *creg1* ^−/−^ mutants and WT siblings at 12–18 hpf (Figure 2C and Figure S2A,B, Supporting Information), indicating the initial erythroid specification was unaffected. These results were further confirmed by quantitative real‐time PCR (qPCR) analysis (Figure 2D and Figure S2C,D, Supporting Information). In contrast, the expression of erythroid differentiation markers *alas2* (an erythroid enzyme catalyzing the first step of heme biosynthesis), *band3* (an erythrocyte membrane protein), and *globins* were strikingly reduced (Figure 2E,F). Moreover, we observed fewer *o‐*dianisidine‐positive erythroid cells in *creg1* ^−/−^ mutants (Figure 2G and Figure S2E, Supporting Information). Thus, these results suggest erythroid specification initiated normally in the mutants, but terminal erythroid cell differentiation was hindered. To further characterize the defective erythroid differentiation in *creg1*‐deficient embryos, we examined the morphology of erythroid cells isolated from Tg (*gata1:dsRed*) zebrafish using Wright‐Giemsa staining. As shown in Figure 2H,I, the erythrocytes of *creg1* ^−/−^ mutants were larger in size and had a higher nucleo‐cytoplasmic ratio than the WT group, reflecting some abnormalities in erythrocyte maturation. Together, our findings demonstrated that the differentiation and maturation of erythrocyte, rather than the specification process, was impeded when *creg1* was depleted. In addition, given the importance of red blood cells in hypoxia tolerance,^[^ ^33^, ^34^ ^]^ we evaluated the hypoxia tolerance of *creg1* mutants to gain insights into the impact of *creg1* on erythroid cell function. The findings revealed that following simultaneous exposure of *creg1* ^+/+^ and *creg1* ^−/−^ larvae to hypoxia (1% O_2_) for 16 hours, *creg1* ^−/−^ larvae exhibited a higher mortality rate compared to *creg1* ^+/+^ larvae. No significant differences were observed between *creg1* ^+/+^ and *creg1* ^−/−^ larvae under normoxia (21% O_2_) (Figure S2F, Supporting Information). This result suggests that *creg1* deficiency impairs erythroid cell function by reducing their tolerance to hypoxia.

To confirm the specificity of phenotypes observed in *creg1*‐deficient embryos, mRNA rescue experiments were performed. The reduced expression level of *habe1* in *creg1* ^−/−^ mutants was significantly restored by injection of a full‐length zebrafish *creg1* mRNA, as well as a deletion mutant lacking the N‐terminal signal peptide, *creg1* ^△20^ mRNA (Figure 2J), suggesting the specific function of Creg1 in erythropoiesis. In contrast, a potential N‐glycosylation site‐defective mutant, *creg1* ^N135A N169A^, failed to rescue the erythroid defects present in *creg1* ^−/−^ mutants, indicating N‐glycosylation is essential for the regulatory role of Creg1 in erythroid cell differentiation. The interaction with the insulin‐like growth factor II receptor (IGF2R) is necessary for the function of CREG1.^[^ ^19^, ^35^ ^]^ Specifically, IGF2R binds preferentially to the glycosylated forms of CREG1.^[^ ^19^ ^]^ In line with these findings, we observed that the interaction of Creg1^N135A N169A^ with Igf2r was sharply attenuated compared with that of WT Creg1 (Figure S3, Supporting Information). This might be one reason for Creg1^N135A N169A^ being unable to restore the erythroid defects in *creg1* ^−/−^ mutants.

### 2.3 Creg1 Deficiency Leads to Increased Apoptosis of Erythroid Cells

**Figure 3:**
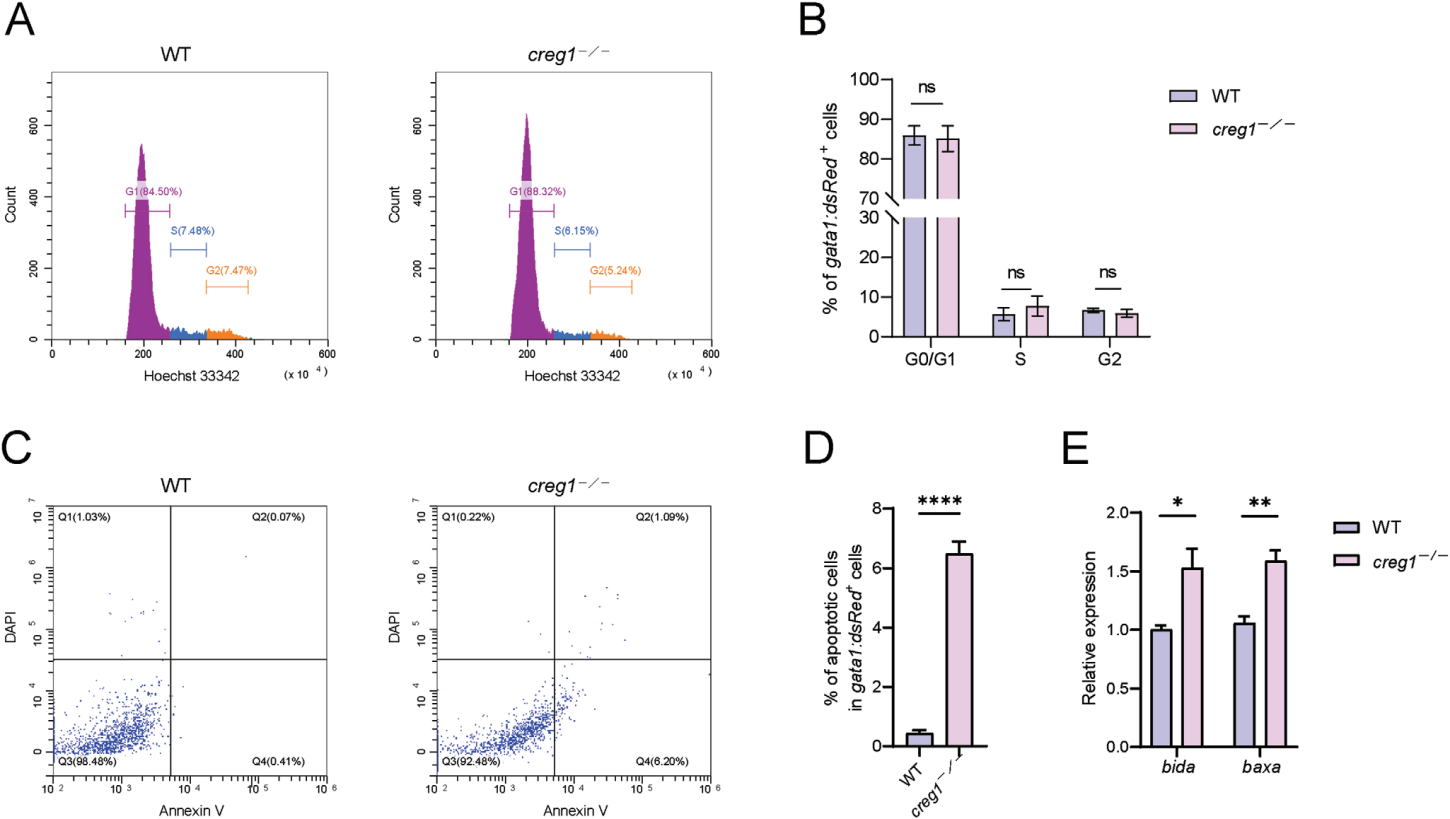
Loss of *creg1* results in elevated apoptosis of erythroid cells. A) Cell cycle analysis of *gata1:dsRed* ^+^ erythroid cells from WT siblings (left) and *creg1* ^−/−^ mutants (right) at 2 dpf. Erythroid cell DNA content was measured by flow cytometry via Hoechst 33342 fluorescence intensity. B) Quantification of the percentage of cells in each cell cycle phase from (A) (*n*=100 embryos per group). C) FACS analyses of apoptotic cell death were performed with *gata1:dsRed* ^+^ erythroid cells from WT siblings (left) and *creg1* ^−/−^ mutants (right) at 2 dpf using Annexin V/DAPI staining. D) Quantification of the percentage of *gata1:dsRed* ^+^ erythroid cells that are Annexin V‐positive from (C) (*n* = 100 embryos per group). E) Quantitative PCR analysis of *bida* and *baxa* expression in *gata1:dsRed* ^+^ erythroid cells from WT siblings and *creg1* ^−/−^ mutants at 2 dpf (*n* = 30 embryos per group). Data shown are the means ± SEM. Statistical significance was calculated using the Student's *t*‐test. *, *p* < 0.05; **, *p* < 0.01; ****, *p* < 0.0001; ns, not significant.

The decreased number of erythrocytes in *creg1* ^−/−^ mutants could be due to reduced cell proliferation and/or increased cell death. We thus first analyzed cell cycle status of *gata1:dsRed* ^+^ erythroid cells at 2 dpf by flow cytometry quantification of DNA content via Hoechst 33342 incorporation. No overt differences in cell cycle arrest were detected between control siblings and *creg1* ^−/−^ mutants (**Figure** **3**A,B). Next, we examined the degree of apoptotic cell death using Annexin‐V/DAPI staining. There was a significant increase in apoptosis in *gata1:dsRed* ^+^ erythroid cells from *creg1* ^−/−^ mutants at 2 dpf (Figure 3C,D). Moreover, we also observed that the expression levels of pro‐apoptotic genes *bida* and *baxa* were remarkably increased in *creg1* ^−/−^ erythroid cells compared to those in the control group (Figure 3E). Together, these data indicate that elevated apoptosis of erythroid cells might lead to reduced numbers of erythrocytes and eventually resulted in anemia in *creg1* ^−/−^ mutants.

### 2.4 Creg1 Regulates Erythropoiesis through the TGF‐β/Smad2 Signaling Pathway

**Figure 4:**
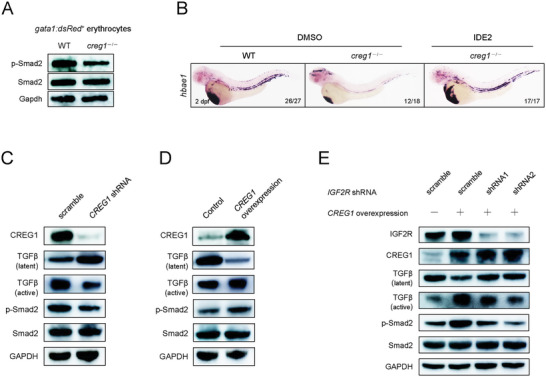
TGF‐β/Smad2 signaling pathway is implicated in *creg1*‐mediated erythroid development. A) Western blot analysis of *gata1:dsRed* ^+^ erythroid cells from WT siblings and *creg1* ^−/−^ mutants at 3 dpf. B) WISH assay of *hbae1* at 2 dpf for embryos treated for 36 h with DMSO or IDE2. C–E) Western blot analysis of TGF‐β/Smad2 signaling‐related proteins in K562 cells.

Previous studies have indicated that the Smad2/3 signaling pathway may serve as a potential regulator of erythropoiesis.^[^ ^36^, ^37^, ^38^ ^]^ In order to explore the mechanistic insights underlying the erythrocytic defect in *creg1* ^−/−^ mutants, we examined whether Smad protein levels were altered in *creg1* ^−/−^ mutants. As shown in **Figure** **4**A, the level of phosphorylated Smad2 (p‐Smad2) was remarkably decreased in erythroid cells sorted from *creg1* ^−/−^/Tg*(gata1:dsRed)* mutants compared to control siblings. To further assess whether the reduced p‐Smad2 contributed to the erythroid defects induced by *creg1* deficiency, we treated embryos with IDE2 which potently activates Smad2 phosphorylation,^[^ ^39^ ^]^ and then determined the effects on erythroid cell differentiation. As expected, IDE2 administration significantly improved the defective erythroid development of *creg1*‐deficient zebrafish compared with DMSO control (Figure 4B). Thus, our results suggest that the dysregulated Smad2 signaling is implicated in the impaired erythropoiesis observed in *creg1* ^−/−^ mutants.

The canonical TGF‐β signaling requires the activation of Smad2/3 by TGF‐β. To investigate the underlying mechanism of how CREG1 activates Smad2, we first examined the TGF‐β expression level. We observed that knockdown of *CREG1* by short hairpin RNA (shRNA) resulted in an increase in the latent TGF‐β protein level and a reduction in its active form in K562 cells (Figure 4C). Reciprocally, overexpression of *CREG1* had the opposite effect (Figure 4D). Similarly, the modulation of TGF‐β expression by CREG1 has also been shown during the differentiation of embryonic stem cells into smooth muscle cells.^[^ ^40^ ^]^ Thus, our data and others indicate that CREG1 activates Smad2 signaling by promoting the conversion of latent to active TGF‐β.

IGF2R, an interaction partner of CREG1, is crucial for the functional role of CREG1.^[^ ^19^, ^35^ ^]^ It has been reported that IGF2R facilitates the activation of latent TGF‐β.^[^ ^41^ ^]^ To determine whether IGF2R mediates the CREG1‐induced TGF‐β/Smad2 signaling activation, we knocked down *IGF2R* expression using shRNA in K562 cells ectopically expressing *CREG1*. The data showed that *CREG1* overexpression led to an increase in the active TGF‐β protein level and a reduction in its latent form. However, these effects were abrogated by *IGF2R* knockdown (Figure 4E), suggesting that IGF2R is necessary for the CREG1‐induced TGF‐β/Smad2 signaling activation.

### 2.5 *Klf1*, a Key Target Gene of Smad Signaling, Is Responsible for the Failure of Erythropoiesis in *creg1* ^−/−^ Mutants

**Figure 5:**
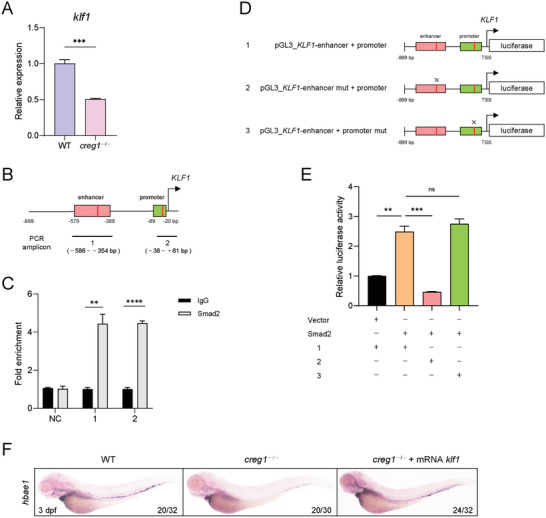
*klf1*, a downstream target gene of the Smad2 signaling pathway, is essential for *creg1* deficiency‐induced aberrant erythropoiesis. A) Quantitative PCR analysis of *klf1* expression between WT siblings and *creg1* ^−/−^ mutants at 3 dpf (*n* = 30 embryos per group). B) Schematic diagram of the *cis*‐regulatory elements of the human *KLF1* locus. Red lines represent positions of potential Smad2 binding motifs. Numbered black lines indicate the position of the amplicons used for PCR quantification of the ChIP signal. C) ChIP‐qPCR analysis using antibodies against IgG or Smad2 was performed in K562 cells. Non‐target region is included as negative control (NC) for specificity of Smad2 enrichment. Data are representative of three independent experiments. D) The structure of recombined firefly luciferase expression vector driven by the *KLF1 cis*‐regulatory elements with normal or mutated Smad2 binding site. E) Luciferase activity assay in 293T cells transfected with Smad2 and distinct reporter constructs. The *Renilla* plasmid was used as an internal control. Data are representative of three independent experiments. F) WISH assay of *hbae1* at 3 dpf. Data shown are the means ± SEM. Statistical significance was calculated using the Student's *t*‐test. **, *p* < 0.01; ***, *p* < 0.001; ****, *p* < 0.0001; ns, not significant.

Klf1 is a well‐studied erythroid transcription factor that coordinates many aspects of terminal erythroid differentiation.^[^ ^42^, ^43^ ^]^ Previous reports have described that sequences located within less than 1 kb upstream of the *Klf1* transcription start site (TSS) are sufficient for erythroid‐specific transcription.^[^ ^44^, ^45^ ^]^ Intriguingly, two potential Smad2 binding sites have been identified within this regulatory region.^[^ ^46^ ^]^ Of note, the transcriptional level of *klf1* was sharply decreased in *creg1* ^−/−^ mutants compared to control siblings (**Figure** **5**A). These prompted us to determine whether *klf1* is a target gene of Smad2 signaling that is responsible for the erythroid defect triggered by *creg1* deficiency. To test this possibility, we first performed chromatin immunoprecipitation (ChIP) followed by quantitative PCR (ChIP‐PCR). The result showed that Smad2 directly bound to the enhancer and promoter region of *KLF1* (Figure 5B,C). Then, we constructed a luciferase reporter vector driven by the *cis*‐regulatory region of *KLF1* harboring the Smad2 binding sites or its mutant form (Figure 5D). The dual‐luciferase assay in 293T cells revealed *Smad2* overexpression significantly increased luciferase transcription driven by the cloned *KLF1 cis*‐regulatory region. However, the mutation of the Smad2 binding site in the enhancer region abrogated the stimulatory effect of Smad2. Intriguingly, the mutation of the Smad2 binding site within the promoter did not affect the stimulatory effect of Smad2 (Figure 5E). These data indicate that Smad2 positively regulates the *KLF1* transcription by binding to its enhancer. Furthermore, *klf1* overexpression significantly restored the defective erythroid development observed in *creg1* ^−/−^ mutants (Figure 5F). Taken together, our findings suggest that *klf1* acts as a target gene downstream of Smad2 signaling that is responsible for the erythroid development mediated by *creg1*.

## 3 Discussion

Although Creg1 has been shown to be involved in several physiological processes, its function in hematopoiesis, particularly erythropoiesis, remains unknown. This work is the first to establish that Creg1 is a critical mediator in erythropoiesis, where *creg1* depletion leads to impaired differentiation and increased cell apoptosis in erythrocytes that contribute to anemia in *creg1* ^−/−^ mutants. Mechanistically, the impact of Creg1 loss on erythropoiesis is likely attributable to reduced activation of TGF‐β/Smad2 signaling pathway and direct downregulation of *klf1* expression. In view of our findings, we propose that Creg1 is a positive regulator of erythropoiesis in part by promoting proper differentiation and survival of erythroid progenitors.

In this study, we found that during zebrafish embryogenesis, *creg1* mRNA was preferentially expressed within the ICM/PBI region where primitive erythropoiesis occurs, indicating its potential role in regulating erythroid development. Moreover, the expression level of *CREG1* increased progressively during erythroid differentiation of HUDEP‐2 and K562 cells. This finding is in agreement with an earlier report, where *CREG1* expression was extremely low in undifferentiated cells but rapidly upregulated upon cellular differentiation.^[^ ^16^ ^]^ Thus, these data indicate that CREG1 may promote cell differentiation in multiple cellular contexts. Supporting this notion, we disrupted *creg1* expression via CRISPR/Cas9 or antisense morpholino oligonucleotides in zebrafish and observed that its loss led to defective terminal erythroid differentiation. While our study primarily focused on erythroid development, we cannot exclude the possibility of CREG1 playing a functional role in other cellular development. Recently, it has been shown that CREG1 deficiency impairs megakaryocyte maturation and platelet production by inactivation of the MEK‐ERK1/2 pathway in mice.^[^ ^47^ ^]^ Additionally, in mouse brown adipose tissue, CREG1 stimulates brown adipocyte formation by binding to retinoid X receptor α.^[^ ^26^ ^]^ CREG1 also promotes cardiomyocyte differentiation and cell‐cell adhesion through its interaction with Sec8.^[^ ^21^ ^]^ Hence, whether the development of these cells was affected in *creg1* ^−/−^ fish need to be further studied in future work.

CREG1 is primarily localized within intracellular compartments but can also be secreted out of the cells.^[^ ^16^, ^35^ ^]^ In the present study, it is unclear whether the defective erythroid development triggered by *creg1* deficiency is due to loss of Creg1 in erythrocytes/progenitors or in the surrounding cells. Of note, we observed that Creg1^△20^, a deletion mutant lacking the secretory signal peptide, could fully restore the erythroid defect in *creg1* ^−/−^ mutants, implying that Creg1 most likely regulates erythroid development in a cell‐autonomous manner. To answer this question more definitively, the development of a mouse line carrying a conditional null allele of *Creg1* in erythrocytes would be envisioned.

TGF‐β signaling pathway is a versatile pathway that regulates cellular processes such as proliferation, differentiation, and apoptosis in both positive and negative directions. It has been reported that TGF‐β inhibits erythropoiesis by blocking proliferation and accelerating differentiation of erythroid progenitors in vitro.^[^ ^48^ ^]^ In addition, overactivation of TGF‐β/Smad2 signaling has been observed in bone marrow precursors of patients with myelodysplastic syndrome (MDS),^[^ ^37^ ^]^ which is characterized by ineffective erythropoiesis. However, our investigations reveal that in *creg1* ^−/−^ mutants, decreased TGF‐β/Smad2 signaling could also lead to anemia caused by defective terminal erythroid differentiation and excessive cell apoptosis, implying that TGF‐β/Smad2 signaling may play a protective role in regulating erythropoiesis under in vivo physiological conditions. This notion is supported by a recent study that inhibition of TGF‐β signaling enhanced rather than suppressed the anemia in *sf3b1* mutants.^[^ ^49^ ^]^ Thus, these findings indicate that the TGF‐β/Smad2 signaling pathway is much more complex than previous thought, and it will be of interest to explore the mechanisms underlying the different roles of TGF‐β/Smad2 signaling in erythropoiesis under both physiological and pathological conditions in future studies.

In summary, our study provides the first genetic evidence that Creg1 plays an active role in erythroid development. We also uncover mechanistic insights into how *creg1* deficiency drives aberrant erythropoiesis in vivo. Moreover, the findings may provide new potential therapeutic targets for improving erythropoiesis in patients with related disorders.

## 4 Experimental Section

### Zebrafish

Zebrafish maintenance and staging were performed as described previously.^[^ ^50^ ^]^ The transgenic line Tg(*gata1:dsRed*) was used in the present study. Zebrafish embryos at 12 hpf were treated with IDE2 (100 µmol) (Biogems, 1139374) or DMSO for 36 h, and then were collected for subsequent analysis. For hypoxia treatments, zebrafish larvae were incubated at 28.5 °C in an Oxycycler (Biospherix)‐controlled incubator. The zebrafish facility and study were approved by the Ethics Committee of Rui Jin Hospital Affiliated to Shanghai Jiao Tong University School of Medicine and the methods were carried out in accordance with the approved guidelines.

### CRISPR/Cas9 Mutagenesis

To generate targeted disruptions in the *creg1* genomic locus, the CRISPR/Cas9 system was employed. Briefly, guide RNA (gRNA) was designed using an online tool ZiFiT Targeter software (http://zifit.partners.org/ZiFiT). Then gRNA was synthesized in vitro with a PCR product‐based approach, and purified using mirVana miRNA Isolation Kit (Ambion, AM1561). Recombinant Cas9 protein (New England Biolabs, M0646T) and gRNA were coinjected into one‐cell stage zebrafish embryos. The injected F0 founders were raised to maturity and then outcrossed with wild type zebrafish to produce F1 embryos. Genomic fragments for the targeted genomic locus were amplified, cloned into the pMD18‐T vector and sequenced to identify potential indel mutations. The F2 mutant line with the homozygous mutation was obtained via incross of F1 heterozygotes. The *creg1* ^−/−^ mutant line was genotyped by Sanger sequencing of PCR fragments covering the gRNA target site.

### Whole Mount In Situ Hybridization

Whole mount in situ hybridization was performed as described previously.^[^ ^51^ ^]^ Digoxigenin‐labeled RNA probes were transcribed with T7 (New England Biolabs, M0251S), T3 (New England Biolabs, M0378S), or SP6 polymerase (Invitrogen, AM1340). The probes were detected using alkaline phosphatase (AP)‐coupled anti‐digoxigenin Fab fragment antibody (Roche, 11093274910) with BCIP/NBT staining (Vector Laboratories, SK‐5400). The stained embryos were then photographed using a stereomicroscope (Nikon) equipped with a digital camera.

### shRNA, Morpholino Oligonucleotide and mRNA Synthesis

The sequences of shRNA were as follows: *CREG1* shRNA, 5′‐CCCATATACATGATTTCAGAA‐3′, *IGF2R* shRNA1, 5′‐CCTGCAAGAAAGACATATTTA‐3′ and *IGF2R* shRNA2, 5′‐AGCGGAGGTTCATCCTATATT‐3′.

MO against zebrafish *creg1* (5′‐CAACATCACGAGCGAGCGGGAGAA‐3′) was designed and purchased from Gene Tools. Zebrafish embryos were microinjected with MO at one‐cell stage, and the dose of injection per embryo was 2.08 ng. Capped mRNAs were synthesized from linearized plasmids using the mMessage mMachine SP6 kit (Invitrogen, AM1340) and diluted to 50–150 ng µL^−1^ for microinjection.

### Cell Culture

293T or K562 cells were cultured in DMEM (Gibco, 11995073) or RPMI (Gibco, 11875119) supplemented with 10% fetal bovine serum (Gibco, 10099141C) at 37 °C in a humidified atmosphere with 5% CO_2_, respectively. Hemin (30 µmol) (Sigma, 51280) was used for erythroid differentiation of K562 cells. HUDEP‐2 cells, provided by the RIKEN BRC through the National BioResource Project of the MEXT, were maintained with StemSpan SFEM II medium (Stem Cell Technologies, 09655) supplemented with dexamethasone (10^−6^ mol), doxycycline (1 µg mL^−1^), EPO (3 IU mL^−1^), and SCF (50 ng mL^−1^). To induce differentiation, HUDEP‐2 cells were cultured in StemSpan SFEM II supplemented with doxycycline (1 µg mL^−1^), EPO (3 IU mL^−1^), SCF (50 ng mL^−1^) and 10% FBS for 5 d and then transferred into StemSpan SFEM II supplemented with doxycycline (1 µg mL^−1^), EPO (3 IU mL^−1^), SCF (100 ng mL^−1^) and 30% FBS for 12 d.

### Wright‐Giemsa Staining and *o*‐Dianisidine Staining

Erythroid cells from zebrafish embryos were sorted and morphologically analyzed using Wright‐Giemsa staining (Baso, BA‐4017). Cytospin preparations of 5×10^5^ cells were incubated sequentially in solution A for 1 min and solution B for 9 min, washed with water, air‐dried, and then examined under a microscope. Images were taken with Zeiss A2 microscope (Zeiss).

*o*‐dianisidine staining was performed as previously described.^[^ ^52^ ^]^ Briefly, dechorionated zebrafish embryos were incubated for 20 min with staining buffer (0.6 mg mL^−1^ *o*‐dianisidine, 10 mmol sodium acetate [pH 5.2], 0.65% hydrogen peroxide and 40% ethanol).

### Plasmid Construction

The zebrafish *creg1*, *igf2r, klf1*, and human *Smad2* were cloned into pCS2^+^ vector. The ‐889 bp upstream of human *KLF1* transcription start site was cloned into pGL3 basic vector. The indicated primers for construction were shown in Table S1 (Supporting Information).

### Cell Cycle and Apoptosis Analysis

Tg(*gata1:dsRed*) and *creg1* ^−/−^/Tg(*gata1:dsRed*) larvae at 2 dpf were dissociated into single cells using 0.25% trypsin (Sigma‐Aldrich, 25200‐056). Single‐cell suspension was obtained by centrifugation at 400 *g* for 5 min, washing twice with PBS, and passing through a 40 µm nylon mesh filter. For cell cycle analysis, cells were incubated with Hoechst 33342 (Thermo Fisher Scientific, H3570) at room temperature in the dark for 30 min. For apoptosis analysis, cells were resuspended in 100 µL binding buffer and stained with 5 µL Annexin V‐FITC (Thermo Fisher Scientific, BMS500FI‐300) for 10 min in the dark at room temperature. Then 1 µL DAPI (Santa Cruz, sc‐3598) was added to the samples immediately before the flow cytometric analysis. Data acquisition was performed on a Beckman CytoFlex S cytometer and analyzed with CytExpert version 2.3 software.

### Co‐Immunoprecipitation (Co‐IP) and Western Blot (WB)

Co‐IP was performed in 293T cells, which were transfected with indicated plasmids. Cells were lysed 48 h later and then lysates were incubated with anti‐FLAG magnetic beads (MedChemExpress, HY‐K0207) for immunoprecipitation.

Western blot analysis was performed using standard methodology with the following antibodies: CREG1 (Thermo Fisher Scientific, PA567717), TGF‐β (Cell Signaling Technology, 3711), IGF2R (ABclonal, A21055), phospho‐Smad2 (Ser250) (Beyotime Biotechnology, AF2545), Smad2 (Abways, CY5593), GAPDH (Proteintech, 60004‐1), HA (Cell Signaling Technology, 3724) and FLAG (Sigma, F‐3165).

### Quantitative Real‐Time PCR (qPCR)

Total RNA was extracted with TRIzol reagent (Thermo Fisher Scientific, 15596026) according to the manufacturer's recommendations. cDNA was synthesized with the RevertAid First Strand cDNA Synthesis Kit (Thermo Fisher Scientific, K1622). qPCR was performed by a qTOWER^3^G (Analytik Jena) using SYBR Green Master Mix Reagent (Yeasen, 11199ES08) following the manufacturer's protocols. Relative expression was quantitated using the ΔΔCt method. The primers for qPCR were shown in Table S1 (Supporting Information).

### Luciferase Reporter Assay

293T cells were transfected with indicated plasmids using Effectene Transfection Reagent (QIAGEN, 301427). Cells were harvested 48 h after transfection and luciferase activities were analyzed using the Dual Luciferase Reporter Assay Kit (Promega, E1910) according to the manufacturer's protocols. Luciferase activity was normalized to *Renilla* activity.

### Chromatin Immunoprecipitation (ChIP) Assay

ChIP assay was performed with SimpleChIP Plus Enzymatic Chromatin IP Kit (Cell Signaling Technology, 9005). Briefly, K562 cells were harvested and crosslinked with 1% formaldehyde for 10 min at room temperature. After sonication, the soluble chromatins were incubated with the following antibodies separately: Smad2 (zenbio, 382472) or control IgG (Cell Signaling Technology, 2729). The immunoprecipitated complex was washed, and DNA was extracted and purified. ChIP DNA was analyzed by qPCR, and the data were normalized by input DNA. The primers used for ChIP‐qPCR are listed in Table S1 (Supporting Information).

### Statistical Analysis

Statistical analysis was performed using Graph Pad Prism software. All values are expressed in mean ± SEM except where noted. A two‐tailed Student's *t*‐test was used for comparisons between the indicated groups studied. *P* value < 0.05 was considered to be statistically significant. The sample size (*n*) for each statistical analysis was provided in the figure legends.

## Conflict of Interest

The authors declare no conflict of interest.

## Author Contributions

X.H., W.X.H., and D.G.L. performed experiments and collected data; X.H.L., J.Z., and H.d.T. provided suggestions on experimental design and data presentation; J.Z. provided suggestions on experimental design and analyzed data; H.Y. designed the research, analyzed data and wrote the manuscript.

## Supporting information

Supporting Information

## Data Availability

The data that support the findings of this study are available from the corresponding author upon reasonable request.
