# Supplementary material for: Creg1 Regulates Erythroid Development via TGF‐β/Smad2‐Klf1 Axis in Zebrafish
Source: Adv Sci (Weinh). 2024 Jul 2;11(33):2402804. doi: 10.1002/advs.202402804 (PMC11434009; doi:10.1002/advs.202402804)
Supplement: Supplementary file 1 — Supporting Information [file ADVS-11-2402804-s001.pdf]

ADVANCED SCIENCE
Open Access

Supporting Information

for *Adv. Sci.*, DOI 10.1002/advs.202402804

Creg1 Regulates Erythroid Development via TGF-β/Smad2-Klf1 Axis in Zebrafish

Xiao Han, Wenxin He, Dongguo Liang, Xiaohui Liu, Jun Zhou, Hugues de Thé, Jun Zhu and Hao Yuan**

Supporting Information

Creg1 Regulates Erythroid Development via TGF-β/Smad2-Klf1 Axis in Zebrafish

Xiao Han, Wenxin He, Dongguo Liang, Xiaohui Liu, Jun Zhou, Hugues de Thé, Jun Zhu* and Hao Yuan*

Supplementary Figure legends

Supplementary Figure S1. Knockdown of *creg1* leads to defective erythropoiesis. (A-B) WISH assay of *hbae1* at 2 dpf in WT siblings and *creg1* morphants. (C-D) *o*-dianisidine staining in WT siblings and *creg1* morphants at 2 dpf.

Supplementary Figure S2. Depletion of *creg1* impairs erythroid differentiation rather than specification. (A-B) WISH assay of *scl* or *gata1* in WT siblings and *creg1*$^{-/-}$ mutants at 14 and 18 hpf, respectively. (C-D) Quantitative PCR analysis of *scl* or *gata1* expression in WT siblings and *creg1*$^{-/-}$ mutants at 14 and 18 hpf, respectively. (E) *o*-dianisidine staining in WT siblings and *creg1*$^{-/-}$ mutants at 7.5 dpf. (F) The survival rate curve of zebrafish larvae exposed to different oxygen concentrations (1% or 21%) at 72 hpf (n = 200 larvae/group).

Supplementary Figure S3. The interaction of Creg1$^{N135A\ N169A}$ with Igf2r was attenuated compared with that of WT Creg1. Western blot of Co-IP analysis in 293T cells expressing HA-Igf2r and FLAG-Creg1 (or FLAG-Creg1$^{N135A\ N169A}$).

Supplementary Figure S1

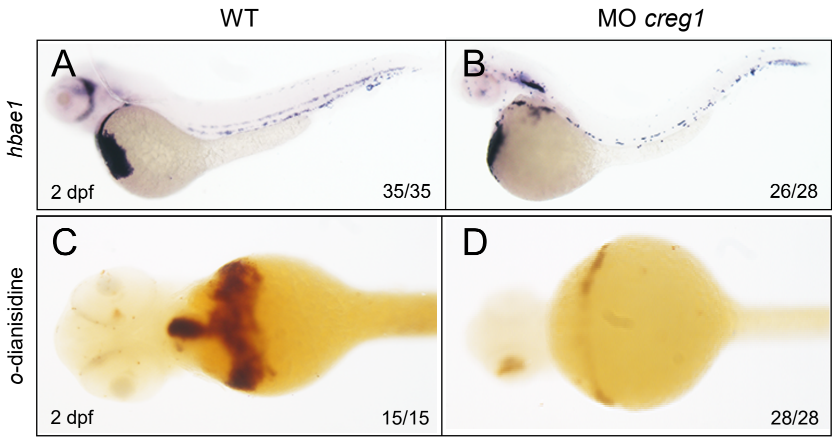

Supplementary Figure S2

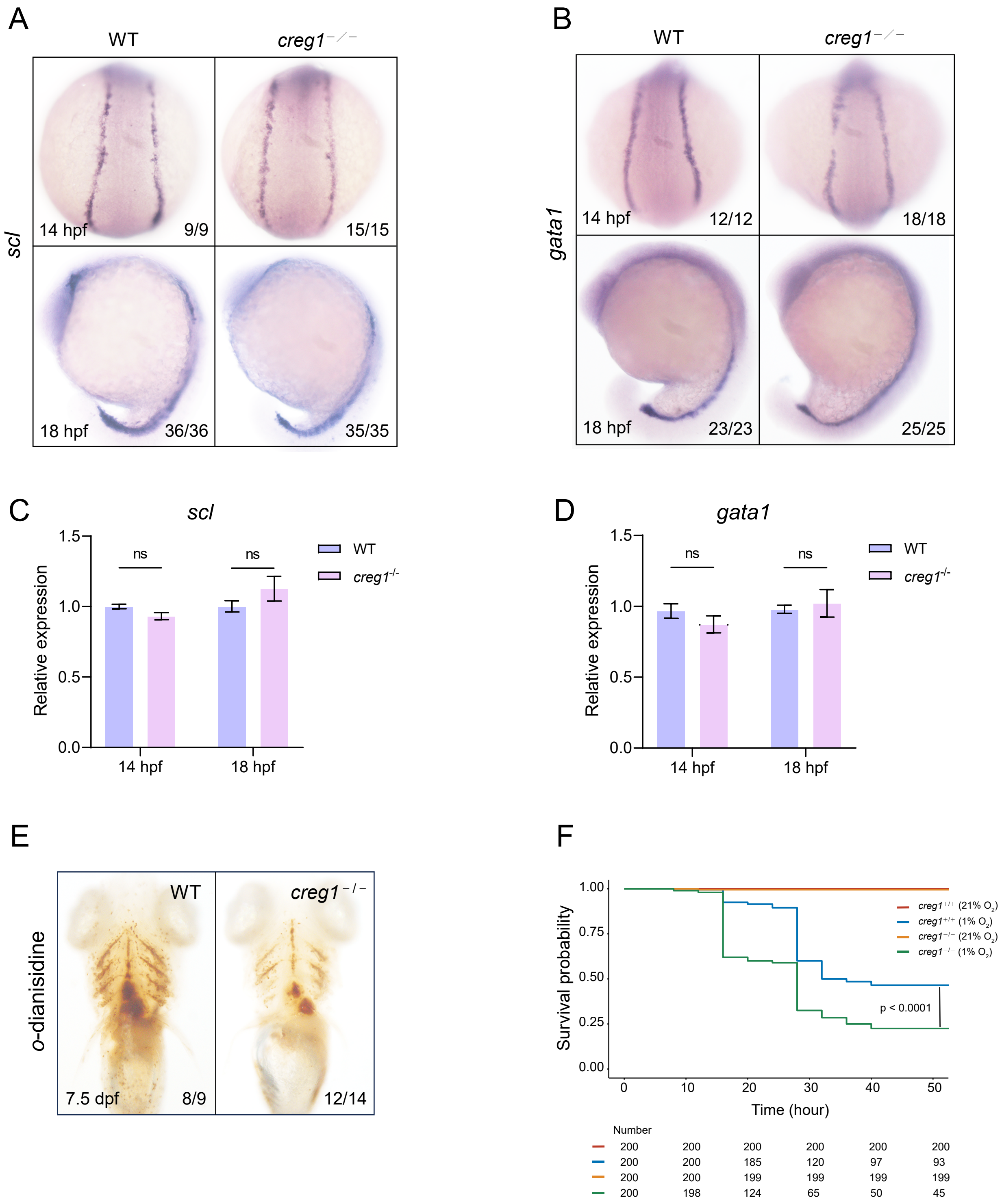

Supplementary Figure S3

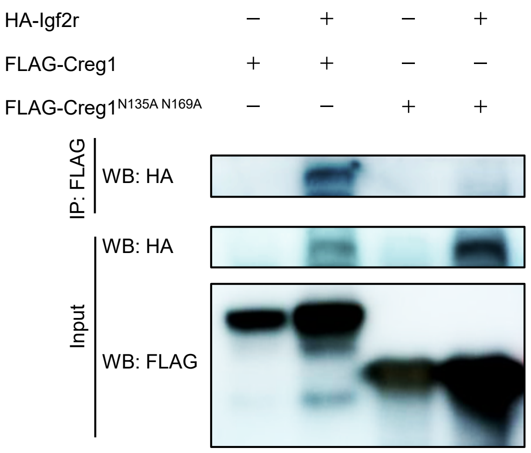

Supplementary Table S1

real-time PCR primers

Gene	Sequence(5'to3')
zebrafish-*hbae1* -qp-FP	CTGAGGCTGTCAGCAAAATCG
zebrafish-*hbae1* -qp-RP	GAACAAAGTGGCCAGAACCAC
zebrafish-*hbae3* -qp-FP	GCTGATGGATGACCTGAAGGG
zebrafish-*hbae3* -qp-RP	CTCAGGAGTGAAGTCGTCTGG
zebrafish-*hbae5* -qp-FP	TGCTGAACCTCAGTGAATTGC
zebrafish-*hbae5* -qp-RP	GGAACTTGTCGATGGCCAGAT
zebrafish-*hbbe1* -qp-FP	TCCACGTAGATCCCGACAAC
zebrafish-*hbbe1* -qp-RP	TACTGTCTTCCCAGAGCGGA
zebrafish-*hbbe2* -qp-FP	GGACTGGACAGAGCCATGAAG
zebrafish-*hbbe2* -qp-RP	GAGGCAATCACGATTGTCAGG
zebrafish-*hbbe3* -qp-FP	TTGTGTGGACAGCTGAGGAG
zebrafish-*hbbe3* -qp-RP	ACGGATAGACGACCAAGCAT
zebrafish-*actin* -qp-FP	TGCTGTTTTCCCCTCCATTG
zebrafish-*actin* -qp-RP	TTCTGTCCCATGCCAACCA
zebrafish-*alas2* -qp-FP	AGGACAACTTTAACCGCCCC
zebrafish-*alas2* -qp-RP	CTCCGCAAAACGGTTCACAG
zebrafish-*gata1a* -qp-FP	TAGACACAGTCCAGTTCGCC
zebrafish-*gata1a* -qp-RP	TACTGGACCAGACCGTGGAT
zebrafish-*klf1* -qp-FP	CAAGCCGTACTGCCTTCATT
zebrafish-*klf1* -qp-RP	TTTGGTGCAGATCCTTGGAG
zebrafish-*band3(slc4a1a)* -qp-FP	TGTTTCATTTGATGGGGACA
zebrafish-*band3(slc4a1a)* -qp-RP	GTGAAGGTCTGTGGCTGCTC
zebrafish-*scl* -qp-FP	TGAAATCAACGATGGTTCGCAG
zebrafish-*scl* -qp-RP	CAGGAGGGTGTGTTGGGATG
zebrafish-*bida* -qp-FP	TCAGCCTGGTCTTTCAGTCAA
zebrafish-*bida* -qp-RP	AGATGACTGGCCCAAACCTG
zebrafish-*baxa* -qp-FP	GTCTTCATCAGAGTGGCCCG
zebrafish-*baxa* -qp-RP	CCCTGGTTGAAATAGCCTTGA
human-*CREG1* -qp-FP	CAGGAACTGTGACCAAGGTGAA
human-*CREG1* -qp-RP	GGTCCACCAAAGTAGTCCAGG
human-*ACTIN* -qp-FP	CCAACCGCGAGAAGATGA
human-*ACTIN* -qp-RP	CCAGAGGCGTACAGGGATAG
human-*KLF1* -ChIP-1-FP	GCAGGGCTGAGACCCTGGGAG
human-*KLF1* -ChIP-1-RP	ACAGCCCTCCCCCTCCCCAGT
human-*KLF1* -ChIP-2-FP	GGACAGAGAGGAGCCCTCGA
human-*KLF1* -ChIP-2-RP	GGCTGGCTGGTGCCCACCCT

construction primers

Gene	Sequence(5'to3')
zebrafish-*creg1* -FP	CGGGATCCATGTTGCGCGCGCTGCTCCC
zebrafish-*creg1* -RP	CGCTCGAGTCACAGCGTGTCACTTCCTC
zebrafish-*igf2r* -FP	GTTCCAGATTACGCTGAATTCATGGGTCGTGTTGGATTTTGCG
zebrafish-*igf2r* -RP	CTATAGTTCTAGAGGCTCGAGTTAAACCTTCAGCAGATCCTCGT
zebrafish-*klf1* -FP	CCGGAATTCGCTGTGACTCAAGCCGTACTG
zebrafish-*klf1* -RP	GCCTCGAGTCACACGTGTCTCTTCATGTG
zebrafish-*creg1* $^{\triangle 20}$ - FP	CCGGAATTCCTGGTTCTGGTTCCGCCGCAC
zebrafish-*creg1* $^{\triangle 20}$ - RP	TCACAGCGTGTCACTTCCTCTCTCGAGCGG
zebrafish-*creg1* ^{N135A} -FP	CTGCTGCAGCTGGCTGACTCTGAGGAG
zebrafish-*creg1* ^{N135A} -RP	CTCCTCAGAGTCAGCCAGCTGCAGCAG
zebrafish-*creg1* ^{N169A} -FP	TTCTCGAAGATCGCCATCACGCAGGTC
zebrafish-*creg1* ^{N169A} -RP	GACCTGCGTGATGGCGATCTTCGAGAA
human-*Smad2* -FP	CCGGAATTCATGTCGTCCATCTTGCCAT
human-*Smad2* -RP	CCGCTCGAGTTATGACATGCTTGAGCAA
